# Post-placental intrauterine device: expanding access to reproductive health in an African province

**DOI:** 10.1080/07853890.2025.2559129

**Published:** 2025-09-17

**Authors:** Maria de Lourdes Paulo Dias, José Paulo Guida, Thuany Bento Herculano, Belmiro Gonçalves Pereira, Fernanda Garanhani Surita

**Affiliations:** ^a^ Departament of Obstetrics and Gynecology, Universidade Estadual de Campinas – UNICAMP; ^b^ Walter Strangway Hospital, Angola

**Keywords:** Postpartum contraception, post-placental IUD, postpartum period, reproductive planning, reproductive health

## Abstract

**Background:**

Angola has scarce availability of contraceptive methods; the use of post-placental copper intrauterine device (PPIUD) is restricted due to lack of training among professionals and knowledge among women. PPIUD is safe, effective, and scalable solution on contraceptive planning.

**Objectives:**

To implement PPIUD in two Angolan hospitals, evaluating the access, acceptability of patients and health professionals, and safety of the PPIUD.

**Methods:**

We performed a prospective study in two Angolan hospitals located in the Province of Cuito, from November 2021 to March 2022, with follow-up durng 45 days after birth. Women were included independently of route of delivery, and excluded if presented anemia, infection, preterm labour, known uterine malformation, fever during labour or rupture of membranes > 24h. Clinical and demographical data was compared between women who retained PPIUD during follow-up.

**Results:**

117 women were invited, and 83 were included (acceptance rate 70.94%),and 81.93% of pregnancies were unplanned. The majority were black (91,6%), had a relationship (59,4%), did not use contraception (63.86%), had 5 or more children (55.42%), and had up to 4 prenatal consultations (70.73%), with an average age of 29.76 (±5.71) years. There were 66.30% vaginal births and 33.70% cesarean sections. Expulsion rate of the PPIUD was 15.66%, most frequently observed after vaginal birth.

**Conclusion:**

PPIUD was well accepted and maintained during the first follow-up assessment. The results support the implementation of PPIUD as feasible, representing an important opportunity to prevent unintended pregnancy by achieving women’s contraceptive needs.

## Short Condensation

In Angola, a study introduced post-placental IUDs in 2 hospitals: 70.94% acceptance, 75.9% retention. Results show high ease of insertion and acceptance by women and professionals. Addressing contraceptive access can empower communities.

## Background

Angola, a country in Sub-Saharan Africa, is the seventh-largest country on the African continent in terms of territorial dimensions. It has an estimated population of 32 million inhabitants, of which approximately 53% are women; of these, half are young people between 20 and 24 years old. The fertility rate is 6.2 children per woman in urban areas; in rural areas, associated with the low level of education, the number of children per woman is even higher.

The difficulty in accessing/seeking reproductive planning may be related to the consequences of the civil war that the country experienced for more than 25 years, which led to a deficit in the construction of schools and health services and a high illiteracy rate. Therefore, unplanned pregnancy remains a public health problem in this sub-Saharan country, where there is a high fertility rate despite limiting socioeconomic conditions [1]. The lack of adequate reproductive planning using reliable methods, such as intrauterine devices (IUD), imposes on women the risks of pregnancies with short intervals, increasing fertility and maternal, neonatal, and infant mortality rates [1].

Reproductive planning reduces not only the rates of unwanted pregnancies but also the consequences of unintended pregnancies on women’s health. It improves a country’s economic performance, women’s empowerment, and families’ quality of life [2]. In Angola, rates of reproductive planning are low (around 13%), and there is a high maternal mortality rate, which was 239/100,000 live births (LB) in 2015 [3]. Women who are from urban areas, living in provincial capitals, married, and with a high level of education are more likely to receive contraception, especially pills and injectables, showing the impact of inequalities in access to reproductive health.

Long-acting contraceptive methods (LARC), such as IUDs, are allowed by health authorities in Angola. However, evidences from other African countries suggest that they are rarely used [4,5], and there is no program to routinely offer post-placental IUD (PPIUD), which is inserted immediately after birth. There are no recent reliable data regarding the acceptance of IUD among Angolan women, with the latest report dating from 1990 [6].

The PPIUD insertion is a safe and highly effective LARC method. This method offers immediate protection against unintended pregnancy without affecting lactation. Numerous studies have demonstrated low complication rates, including minimal risk of infection or uterine perforation. The expulsion rate is slightly higher when compared to interval insertions. However, the increased contraceptive uptake and reduced risk of short interpregnancy intervals make it a valuable option in comprehensive postpartum care [7].

This study aimed to present the results of a pioneering strategy for training local health staff to offer and insert PPIUD and to assess the acceptance, access, safety, and rate of continued use among women in Bié Province, Angola.

## Methods

**Figure 1.**
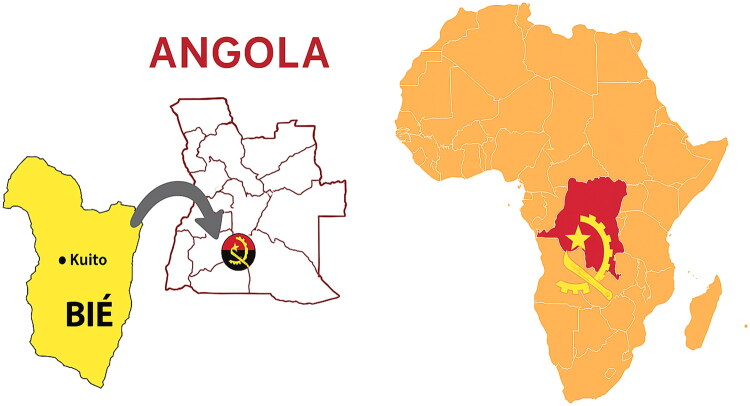
Location of the city of Kuito, Bié Province, in Angola.

We performed a prospective observational cohort study in two hospitals in Kuito (Maternal and Child Center, and Walter Strangwy Hospital). Kuito is a city located in the Bié Province (Angola). The province has a reproductive planning rate of around 2%, a fertility rate of 8.6 children per woman, and a malnutrition rate of 51% [3]. Bié is located in the country’s central region, with an approximate population of 1,654,744 inhabitants, and the predominant ethnic group is Umbundu (Figure 1) [8].

The Walter Strangwy Hospital and the Bié Provincial Health Department, responsible for the Maternal and Child Center, approved this study. All participants signed an informed consent form before participating in the study.

The study was performed from November 2021 to March 2022. The sample was chosen for convenience, based on the availability of IUDs. A total of 83 IUD were available for this study, and the sample size was defined according to this availability. When all IUDs were used, study enrollment was stopped. All the IUDs were provided by the Provincial Health Office in Bié, and were provided to the participants with no costs to them.

The Maternal and Child Center is a secondary health unit, with limited resources to provide care during delivery, and is not able to perform surgical procedures. It is in the center of the city of Kuito, and it performs an average of 260 vaginal births per month. At the same time, the Walter Strangway Hospital is a tertiary hospital performing an average of 190 cesarean sections and 300 vaginal deliveries per month. It has 250 beds and is the only hospital with an operating room in the province. It is the only hospital that performs general obstetric and gynecological surgeries, such as the repair of vesicovaginal fistulas due to obstetric causes, curettage, and tubal ligation. Women in the need of a cesarean section are transferred from the Maternal and Child Center to the Walter Strangway Hospital.

One of the researchers (MLPD), an Angolan doctor specialist in Gynecology and Obstetrics, was trained in a Brazilian obstetrics referral center where PPIUD has already been implemented in clinical practice. After this training, the researcher implemented it in the above-cited Angolan centers (the Maternal and Child Center and the Walter Strangway Hospital). All PPIUDs insertions in Angola were performed under the supervision of that researcher. The Provincial Health Office in Bié provided IUDs. During the study period, the researcher also went to the antenatal care (ANC) clinic of the Maternal and Child Center to present to women and healthcare professionals the advantages of the IUD, and to inform the women that the method was safe and effective, and it would be available in both settings.

The eligible pregnant women were contacted and invited to participate in this study by the researcher responsible for implementing this study in Angola (MLPD). The women were assessed during hospital admission for childbirth. Explanations were provided about the method, its benefits, and the objective of the study. After the explanation, those who agreed to participate in the study signed the consent form. Immediately after birth, they were asked again about their desire to insert PPIUD, and once they confirmed their desire, we proceeded with insertion. The participation in this study was voluntary. The consent form was written in Portuguese, the local language of Angola.

The inclusion criteria for PPIUD insertion were women admitted for birth, independently of the route indicated for birth, and hemoglobin levels >8.0 g/dL during antenatal care (ANC). PPIUD was not offered to women with a diagnosis or suspicion of ovular infection, diagnosis of any other infection (at any site), spontaneous preterm labor, active sexual infection, uterine malformation, fibroids distorting the uterine cavity, fever during labor or birth, rupture of membranes for more than 24 h, manual extraction of the placenta, postpartum hemorrhage or uterine atony. Primigravidae were also excluded due to local cultural aspects.

For women who underwent vaginal birth, a manual technique was used to insert the PPIUD. The first step was to perform asepsis of the vulva, vagina, and cervix with an aqueous chlorhexidine solution. The uterus was palpated to assess the fundus and tone. The IUD was clamped between the index and middle fingers and gently inserted into the uterine fundus. For women who underwent cesarean-section, the uterine cavity was assessed after complete placental expulsion; the uterine fundus was palpated, and after grasping the IUD between the index and middle fingers, it was gently released into the uterine fundus. The IUD strings were guided toward the cervix with the aid of Kelly’s forceps. In both situations, the PPIUD was inserted by the medical team under the supervision of the researcher.

The researcher responsible for the study trained the local staff in PPIUD insertion. The training initially included theoretical instructions on insertion, followed by observation of PPIUD insertion in the first study participants. Subsequently, the local staff began to perform PPIUD insertion independently, but always under the supervision of the researcher responsible. The objective of this training model was to ensure that IUD implementation would be sustainable in the future, allowing the supply to be maintained. After inserting the IUD, professionals filled out a difficulty scale that ranged from 1 to 10, considering difficulty as low as 1–3, moderate as 4–6, high as 7–9 and very high 10.

Upon discharge, patients were scheduled to return to the hospital to coincide with the date of their baby’s vaccination (around 14 days postpartum). Upon return, a transvaginal ultrasound (TVUS) was performed to assess the IUD. An IUD was considered inadequately positioned if it was not located entirely in the endometrial cavity, rotated inversely, or if one of the rods penetrated the myometrium. If IUDs were not visible on TVUS, a simple abdominal and pelvic X-ray was requested as a complementary examination; if IUD was not visible in these two imaging methods, it was considered expelled. In both conditions (inadequate position or expelled), another contraceptive method was provided. For all women, another visit was scheduled for approximately 45 days postpartum.

The data of the included participants were collected using a structured questionnaire designed explicitly for this study. Sociodemographic, obstetric, gynecological, and sexual and reproductive variables were assessed. The data obtained was included in a database specifically built for this study. This database contained the anonymized data from the included participants.

We described frequencies and percentages for the categorical variables. We also compared the following variables: mode of birth, age, parity, interpregnancy interval, among women who expelled the PPIUD with women who retained it, using Chi-square and Fisher’s exact test. The significance level adopted for statistical tests was 5%, and we also estimated the risk ratio (RR) and respective 95% confidence interval (CI). The STROBE (Strengthening the Reporting of Observational Studies in Epidemiology) recommendations were followed [9].

The study protocol was previously approved by the local Ethics board of the Walter Strangway Hospital and the Maternal and Child Center. The inclusion in the study occurred after each participant provided written consent. The principles of the Declaration of Helsinki were followed during this research.

## Results

From November 2021 to March 2022, 117 women were invited, of whom 83 were included after accepting to participate. Among the excluded women, 19 declined participation, and 15 were excluded due to lack of partner consent. Of the women included, 55 (66.3%) had vaginal births and 28 (33.7%) underwent cesarean sections.

The average age of the women included was 29.8 years (+5.7). The majority were Black (91.6%), in a relationship (59.0%), and 49.4% had completed secondary school or more. These data are detailed in Table 1.

**Table 1.** Sociodemographic characteristics of participants with post-placental insertion of the copper-bearing intrauterine device (PPIUD) in the hospitals of Bié, Angola.

Characteristics	Frequency	(*N* = 83)	Percentage (%)
Age (years)			
	20–24	20	24.1
	25–29	14	16.9
	30–34	32	38.5
	≥35	17	20.5
Skin color			
	Black	76	91.5
	Mixed	6	7.3
	White	1	1.2
Partner			
	Without partner	34	41.0
	With partner	49	59.0
Schooling (years)			
	0–6	10	12.0
	7 − 9	32	38.6
	10 − 13	38	45.8
	≥13	3	3.6
Menarche (years)			
	≤12	24	28.9
	>12	59	71.1
First sexual intercourse (years)			
	13	5	6.1
	14	31	37.3
	15	31	37.3
	16	14	16.9
	17	2	2.4
Prior use of contraception			
	Injectable	9	10.8
	Oral	19	22.9
	Subdermal implant	2	2.4
	None	53	63.9
Planned pregnancy			
	No	68	81.9
	Yes	15	18.1
Number of ANC appointments			
	1–2	9	11.0
	3–4	49	59.7
	5–6	25	29.3
Previous births			
	1–2	5	6.1
	3–5	44	53,0
	> = 6	34	40,9
Previous cesarean			
	Yes	26	31.3
	No	57	68.7
Previous miscarriage			
	Yes	24	28.9
	No	59	71.1

Regarding gynecological and obstetric history, most participants reported first sexual intercourse before the age of 18, with the majority reporting very early onset (by age 15) (80.7%). Additionally, 63.9% did not use contraceptive methods, and 81.9% reported the pregnancy as unplanned. High parity and a low number of antenatal care (ANC) consultations were also observed, as shown in Table 1.

In terms of knowledge about PPIUD, 54 women (65.1%) reported learning about the method through friends. In contrast, 24 (28.9%) learned about it through an educational activity conducted by nurses prior to ANC consultations at the Maternal and Child Center. Among the participants, 40 (48.2%) accepted the use of the PPIUD because they considered it a good contraceptive method, and another 27 (32.5%) because it is a long-acting reversible contraceptive (LARC).

Most healthcare professionals reported low (51.8%) or moderate (41.0%) difficulty in performing the procedure. Based on ultrasound and radiological evaluation of the PPIUD 15 days postpartum, 63 (75.9%) were correctly positioned, 13 (15.7%) had been expelled, and 7 (8.4%) were found to be misplaced. These data are presented in Table 2.

**Table 2.** Reproductive planning, reasons for choice, professional barriers, and final status of postplacental copper IUD (PPIUD) use in Bié Hospitals, Angola.

Guidelines and reasons		*N* = 83	%
Prior guidance			
	Yes	37	44.6
	No	46	55.4
Reason for choice			
	Good contraceptive	40	48.2
	Long action	27	32.5
	No cost	7	8.4
	Reversible	9	10.8
How did you hear about PPIUD?			
	Friends	54	65.1
	Nurses	24	28.9
	Television	5	6.0
Difficulty in inserting PPIUD			
	Low	43	51.8
	Moderate	34	41.0
	High	6	7.2
PPIUD final condition			
	Expulsion	13	15.7
	Misplaced	7	8.4
	Well allocated	63	75.9

We compared women who experienced PPIUD expulsion with those who retained the device at the 45-day postpartum evaluation. Among the variables assessed, we observed that the relative risk (RR) of expulsion for women aged ≥35 years was 4.529 (95% CI: 1.749–11.726; *p* = 0.003), and for those with parity ≥6, the RR was 3.243 (95% CI: 1.086–9.679; *p* = 0.033). No significant differences were observed for the other variables between the two groups. These results are presented in Table 3.

**Table 3.** Characteristics of women who expulsed the PPIUD in comparison with those who retained the device in a study in Angola.

Variables		Expulsion *N* = 13	Not Expulsed *N* = 70	Risk ratio (RR)	Confidence interval (CI)	*p* value
Mode of birth					0.84–44.63)	0.051
	Vaginal	12 (92.3)	43 (61.4)	6.11		
	Cesarean	1 (7.7)	27 (38.6)	Ref		
Age (years)					**1.75**–**11.73)**	**0.003**
	≥35	7 (53.8)	10 (14.3)	**4.53**		
	<35	6 (46.2)	60 (85.7)	Ref		
Parity					**1.09**–**9.68**	**0.033**
	≥6	9 (69.2)	25 (35.7)	**3.24**		
	<6	4 (30.8)	45 (64.3)	Ref		
Interpregnancy interval (months)					0.63–33.40	0.099
	<18	12 (92.3)	48 (68.6)	4.60		
	≥18	1 (7.7)	31.4)	Ref		

## Discussion

### Findings and interpretations

This study aimed to evaluate the acceptance, access, safety, and continuation PPIUD use among postpartum women in a province of Angola, Africa. The study also assessed the training strategy for PPIUD insertion.

We found a high acceptance rate of the insertion and a high rate of well-located PPIUDs, among women in Bié, Angola. Considering the social vulnerability of these women, who are mostly young and have many children, offering LARC contraception is even more relevant, especially in a setting where maternal, neonatal, infant, and juvenile mortality rates are extremely high, can contribute to reducing the dimension of the problem [10].

We also found that healthcare professionals showed good acceptance of the method following supervised training, suggesting that the intervention could be sustained if PPIUD availability is ensured. These findings highlight the importance of maintaining effective public health interventions, as is the PPIUD, through adequate resources, given the high acceptance of the method among patients and healthcare providers.

### Research in the context of what is known

A study conducted in Sub-Saharan Africa, in Malawi, including ten hospital centers, concluded that women accepted IUD insertion immediately after delivery in 100% of cases; however, the major difficulty was the lack of trained professionals to follow up on these patients and continue the insertion practice [11]. Furthermore, most professionals who inserted the PPIUDs reported low or moderate difficulty with insertion. Our results support that training professionals to insert PPIUD is viable and demands a low amount of resources to be implemented.

Primigravidae were excluded due to historical complications with the use of the Lipps loop IUD, which brought a stigma to the use of other IUDs due to the risk of infertility, in a place where the cultural background encourages women to have large families, with 4–6 children in average. On the other hand, a woman without children in Angolan culture is not valued; she suffers social pressure, especially in rural areas, where the practice of dowry is still frequent; the pressure comes from the family of the husband who gave the dowry and from the maternal family.

Although this was the first time the PPIUD was offered free to the user in that location, women showed a willingness to use the method, as it is a long-lasting and reversible method. Many women knew friends who used this method, which was purchased in pharmacies and required extra payment for insertion in clinics; others purchased and inserted the IUD during the family planning consultation at the Maternity and Child Hospital, but this is not always accessible.

The results obtained in our study are similar to those of another study conducted in other African countries (Senegal, Ivory Coast, Togo, Benin, Niger and Chad) [12]. The provision of IUDs should be associated with training of professionals, supervision of staff and monitoring of results, which was also carried out in our study. Interest in IUDs appears to be high, with good acceptance of this method in the immediate postpartum period, and with lower expulsion rates than those observed in our study. Increased availability appears to increase its acceptance, which was not possible to demonstrate in our study, due to its duration.

### Clinical implications

Most women did not plan their pregnancy (81.9%), which worsens the gestational outcome, whether due to late discovery of the pregnancy, fewer ANC consultations, previously uncontrolled comorbidities, or clinical or obstetrical conditions not prevented promptly during pregnancy. Unplanned pregnancy increases maternal and perinatal risks of morbidity and mortality [13–15]. Thus, offering a long-lasting reproductive planning method contributes to reducing the complications associated with unplanned pregnancies.

The first sexual intercourse occurred between 13 and 15 years of age in 80.7%; most of these adolescents did not use contraception (63.9%), with a higher risk of becoming pregnant early in life, increasing the risks of dropping out of school, having an increased number of pregnancies during their lifetime, complications during childbirth such as vesicovaginal fistulas and family vulnerability, due to economic fragilities and the need to feed many children [16–18].

A systematic review comparing the insertion of the PPIUD after vaginal birth and after cesarean section between young and older women found that younger women expelled more compared to older women, a result opposite to that found in this study, where the women who expelled the IUD were older. This is probably due to the high number of pregnancies, which are higher as the woman ages. As for the route of birth, as seen in this study, there was more significant expulsion in insertion after vaginal birth compared to cesarean section [19].

A study conducted in Uganda, with similar methods to our research, but a slightly higher number of participants, showed an expulsion rate of 7.8%, lower than that observed in our study (15.7%). The study in question did not evaluate the positioning of the PPIUD, as ours did, which showed that 8.4% were mispositioned. Regarding risk factors, the study did not show age or parity as a risk factor for IUD expulsion. On the other hand, the time of insertion and duration of lochia were associated with a higher risk of expulsion [20].

The expulsion rates of PPIUD observed in other studies ranged from 0% to 46.7%, as previously reported by a systematic review. These rates were higher than those observed in interval IUD. Women who desire PPIUD must be counseled regarding the risk of expulsion, and the need for adequate follow-up to guarantee efficacy of the method. On the other hand, the offer of PPIUD provides an essential window of opportunity to give a safe contraceptive method [19,21].

### Research implications

We compared the women according to the expulsion or not of the IUD during the follow-up. The variables that presented significant differences were maternal age greater than or equal to 35 years and parity greater than or equal to 6. Regarding the mode of birth, vaginal birth showed a non-significant difference, but a limit, for a greater risk of expulsion.

Ultrasound control was an extra precaution to get to know the result more closely, since it is not a universal recommendation and does not need to be performed if this is a barrier to implementing the use of post-placental insertion IUD [22].

The insertion of the IUD is a window of opportunity for contraception, because, due to different factors, women do not return to postpartum consultation. According to a study conducted in Australia, 50% of women who agreed to have the IUD inserted during their postpartum check-up did not attend the postpartum consultation, and only 25% did [15]. A maternity hospital for high-risk pregnancies in Brazil reported a 37% absence, and the absence was associated with worse social conditions and the fact of having other children, a condition present in all women in this study [23].

Our study did not evaluate specifically the uptake rate since we had a limited availability of PPIUD, and enrollment was stopped when the total number of PPIUDs was reached. Among the 117 invited participants, 19 (16.2%) declined to participate and 15 (12.8%) were not allowed by their partners. This result suggests a high uptake rate—the main concern is regarding the availability of the method. The lack of availability is probably the main gap in the uptake rate of PPIUD—and providers and health planners must remove unnecessary barrier to the access to this contraceptive method [24].

The rates of reproductive planning coverage in Angola are low, currently at 13% [3]. Strategies such as the one presented in this study can encourage local governments to disseminate LARCs to reduce maternal morbidity and mortality, increase the interpregnancy interval, and reduce the fertility rate. The Angolan provinces of Cuando, Cubango, and Muxico present a coverage rate of around 1%, and are in the most urgent situation regarding this need.

### Policy implications

Our results show high acceptance of the IUD, consistent with other results obtained in countries with similar development indices [25–27]. Training professionals for insertion did not require expensive investments for low-income countries. Our study cannot show how many unwanted pregnancies will be preventable. Still, considering the effectiveness of the IUD and its low expulsion rate, it will likely be a considerable number. Therefore, health services must make available several contraceptive methods, including the IUD. This is a safe, inexpensive, highly effective method, and its availability for insertion immediately after birth allows several barriers to returning to the health service to be overcome. Therefore, we advocate that its availability be considered in a context of regular and universal provision for all women who desire it, especially in low- and middle-income countries.

### Strengths and limitations

Our study has some limitations, such as the small number of participants, on the other hand, it presented the results of implementing PPIUD in a low-income country, showing high rates of acceptance and continuity of the method. Another limitation is the follow-up period is only up to 45 days; however, studies have shown that the period of greatest risk of exposure is after 30 days. A randomized clinical trial conducted in Brazil, at the same training site as the first author, followed women who had PDI inserted for up to one year, and showed that expulsions occurred before the 42-day postpartum visit; the one-year follow-up showed that the vast majority of expulsions occurred early [28,29]. The long-term follow-up of the included women may provide more evidence regarding the efficacy of the proposed strategy.

## Conclusions

Offering PPIUD is an opportunity for long-term, reversible contraception with high continuity and was very well accepted among the women and considered an easy procedure by health professionals in this study in Bié, Angola. Its impact is even greater in a population with low access to sexual and reproductive health, thus contributing to reducing the rate of unplanned pregnancies, increasing the interval between pregnancies, thus reducing the rate of maternal, neonatal and infant mortality, and guaranteeing sexual and reproductive rights.

## Data Availability

Data is available through reasonable request to the corresponding author.

## Paper context

Main findings: The postpartum intrauterine copper device was highly accepted, and the majority of included women remained with it after 45 days of insertion.

Added knowledge: the postpartum intrauterine copper device is culturally accepted by women in Angola – a country with high rates of unintended pregnancies and maternal and perinatal mortality.

Global health impact for policy and action: the implementation of postpartum intrauterine copper device is feasible in low-resourced countries, which may guide policies in other similar countries in Africa, Asia and Latin America.
